# Short-chain fatty acids in breast milk and their relationship with the infant gut microbiota

**DOI:** 10.3389/fmicb.2024.1356462

**Published:** 2024-02-19

**Authors:** Menglu Xi, Yalu Yan, Sufang Duan, Ting Li, Ignatius Man-Yau Szeto, Ai Zhao

**Affiliations:** ^1^Vanke School of Public Health, Tsinghua University, Beijing, China; ^2^Inner Mongolia Yili Industrial Group Co. Ltd., Yili Maternal and Infant Nutrition Institute (YMINI), Beijing, China; ^3^Inner Mongolia Dairy Technology Research Institute Co. Ltd., Hohhot, China; ^4^National Center of Technology Innovation for Dairy, Hohhot, China

**Keywords:** short-chain fatty acids, breast milk, whole genome shotgun sequencing, infant microbiota, KEGG

## Abstract

**Introduction:**

The short-chain fatty acids (SCFAs) contained in breast milk play a key role in infant growth, affecting metabolism and enhancing intestinal immunity by regulating inflammation.

**Methods:**

In order to examine the associations between the microbiota and SCFA levels in breast milk, and explore the roles of SCFAs in regulating the infant gut microbiota, we enrolled 50 paired mothers and infants and collected both breast milk and infant fecal samples. Breast milk SCFA contents were determined by UPLC-MS, and whole genome shotgun sequencing was applied to determine the microbial composition of breast milk and infant feces. The SCFA levels in breast milk were grouped into tertiles as high, medium, or low, and the differences of intestinal microbiota and KEGG pathways were compared among groups.

**Results:**

The results demonstrated that breast milk butyric acid (C4) is significantly associated with Clostridium leptum richness in breastmilk. Additionally, the specific *Bifidobacterium* may have an interactive symbiosis with the main species of C4-producing bacteria in human milk. Women with a low breast milk C4 tertile are associated with a high abundance of *Salmonella* and *Salmonella enterica* in their infants' feces. KEGG pathway analysis further showed that the content of C4 in breast milk is significantly correlated with the infants' metabolic pathways of lysine and arginine biosynthesis.

**Discussion:**

This study suggests that interactive symbiosis of the microbiota exists in breast milk. Certain breast milk microbes could be beneficial by producing C4 and further influence the abundance of certain gut microbes in infants, playing an important role in early immune and metabolic development.

## Introduction

Breast milk is rich in nutrients and is considered as the most ideal natural food for infants aged 0–6 months (Gates et al., 2023; Szugye et al., 2023). Not only it can ensure infants' growth; certain breast milk components also play essential roles in regulating intestinal microbiota colonization and metabolism; serve as anti-inflammatory and anti-infectious agents; and perform many other functions (Fougère et al., 2023; Ouyang et al., 2023; Ozen et al., 2023).

Fat is the second most abundant solid nutrient in breast milk and can provide around 50% of the energy needs of newborns (George et al., 2021). In addition to its richness in medium- and long-chain fatty acid content, breast milk also contains small amounts of free short-chain fatty acids (SCFAs, including acetic acid C2, propionic acid C3, butyric acid C4, isobutyric acid iC4, 2-Methylbutyric acid mC4, valeric acid C5, and isovaleric acid iC5) (Stinson et al., 2020; Stinson and Geddes, 2022). The level of SCFAs in breast milk varies greatly among individuals. Different studies have found that the total content of SCFAs in breast milk ranges from 13 to 4,300 μmol/L (Prentice et al., 2019; Stinson et al., 2020). However, the source of breast milk SCFAs is still not clear. Some studies indicate that breast milk SCFAs may be produced by the maternal gut microbiota and distributed to the breast through circulation; however, it is also suggested that they could be produced in breast milk by its own microbiota (Stinson et al., 2020; Stinson and Geddes, 2022). Currently, increasing evidence demonstrates that SCFAs play key roles in infant growth and development (Nogal et al., 2023). *In vitro* and *in vivo* studies have found that the breast milk SCFAs could help to decrease inflammation, increase immunity, and reduce intestinal inflammation (Gao et al., 2021; Xu et al., 2023). In addition, breast milk SCFAs could affect in infant metabolism by regulating inflammation. One study showed that breast milk SCFAs, especially C4, can directly or indirectly affect fat formation and fat cell metabolism through anti-inflammatory mechanisms in infants (Prentice et al., 2019). Furthermore, C4 is reported primarily associated with the growth of intestinal epithelial cells and could benefit for the development of immunity (Meijer et al., 2010; Brahe et al., 2013). However, the previous studies mainly conducted *in vitro* cell tests and animal experiments, and the number of population-based study is limited. One population-based cohort study revealed that breast milk SCFAs, including C2, C3 and C4, as the essential energy sources could protect essential fatty acids from oxidation, which could regulate infant growth parameters and bring a protective effect against overweight in early life (Prentice et al., 2019). This finding suggested specific breast milk SCFAs may have certain functions in early life. However, the research on specific breast milk SCFAs and their impact on infants is quite limited, with the exploration of potential regulatory mechanisms being rarely undertaken.

It is well known that certain microbials can produce SCFAs in the gut (Shi et al., 2023). Likewise, it has been suggested that one of the functions of SCFAs is to regulate the gut microbiome (Gotoh and Shibata, 2023). A previous study found that SCFAs, in particular C4, can inhibit the growth of *Escherichia coli* or *Salmonella in vitro*. By nourishing intestinal cells and inhibiting the spread of *Salmonella* in the intestine, butyrate could significantly enhance colon epithelial barrier function (Homann et al., 2023). However, to our knowledge, there are no data reported on the associations between breast milk SCFAs and the infant microbiota. Therefore, whether breast milk SCFAs could affect infant health in the same way as those produced by gut microbes should be examined, and whether the health functions of SCFAs rely on its regulating roles in the infant gut microbiota is worth exploring.

Using paired mother-infant samples, this pilot study aims to (1) examine the associations between the microbiota and SCFA levels in breast milk, and (2) explore the roles of breast milk SCFAs in regulating the infant gut microbiota and its related metabolic functions.

## Materials and methods

### Participants

A total of 50 paired mothers and their infants were recruited from three Chinese cities (Xuchang, Cangzhou, and Chenzhou), and breast milk and infant fecal samples were collected. The current study design included mothers who were at 1–3 months postpartum with (1) a healthy singleton birth, (2) a full-term delivery (37–42 weeks), and (3) exclusive breastfeeding. Their paired infants should have (1) no disability or disease diagnosed at birth and (2) an Apgar score ≥8. Mothers with gastric and intestinal illnesses and nipple or lacteal gland diseases were excluded. Infants who had received any prebiotics, probiotics, or antibiotics in the last 4 weeks prior to sample collection were excluded from the current analysis. All participants provided written informed consent. The protocol was approved by the Research Center for Public Health, of Tsinghua University (No. THUSM/PHREC/2021-003).

### Sample collection

The study was conducted by trained health professionals using interview questionnaires to collect relevant information about the subjects, including sociodemographic characteristics, lifestyle, pregnancy information, and birth history. The height and weight of lactating women were measured by trained professionals at clinics.

Breast milk was sampled using a standardized method for all subjects to avoid the influence of related factors on the composition of breast milk. The protocol was as follows: nursing mothers were instructed to feed their infants, empty their breasts between 6 and 7 a.m., and collect breast milk between 9 and 11 a.m. Whole milk (fore and hind milk) was collected from one breast using a sterile breast pump. The breast milk was gently mixed and then immediately frozen at −80°C. Around 3 g of infant feces was collected using a sterile collection tube. Samples were stored immediately at −80°C.

### SCFA analysis

SCFAs were extracted from human milk using acetonitrile and derivatized using 3-nitrophenylhdyrazones. SCFAs were analyzed on a Jasper HPLC coupled to a Sciex 4500 MD system. Individual SCFAs were separated on a Phenomenex Kinetex C18 column (100 × 2.1 mm, 2.6 μm) using 0.1% formic acid in water as mobile phase A and 0.1% formic acid in acetonitrile as mobile phase B. Octanoic acid-1-13C1 purchased from Sigma-Aldrich and Butyric-2,2-d2 from CDN Isotopes were used as internal standards for quantitation.

### Extraction and whole genome shotgun sequencing

A QIAamp DNA Stool Mini Kit (QIAGEN, Hilden, Germany) was used to extract DNA from samples. The extracted DNA was tested for its concentration (≥12.5 ug/ul), integrity (the main peak of the electrophoresis glue map > 20 kb) and purity (no protein, RNA/salt ion contamination). Samples meeting the above conditions entered the library construction process. Library preparation and subsequent metagenomic sequencing were carried out on the Illumina's novaseq PE150, and the sequencing technology was whole genome shotgun sequencing.

### Data processing and analysis

The raw fastq files were demultiplexed based on the indices. The raw data were trimmed and quality controlled using trimmomatic (Version 0.35) and cutadapt (Version 1.16) software. The host sequences were removed by using bowtie2 (Version 2.2.5) aligned with GRCh38/hg38 sequence. The clean data were assembled and predicted ORF by software Megahit (Version 1.2.9) and Prodigal (Version 2.6.3). The predicted genes were clustered and a non-redundant gene catalog was constructed with the CD-HIT (Version 4.8.1) (parameters: identity = 95%, coverage = 90%). The abundance of genes was estimated by the genomeCoverageBed in Bedtools (Version 2.25.0). Then the gene abundance was normalized to 1 MB. The representative sequences for each ORF were assignment for taxonomy against NCBI nr database with a cut-off E-value of 1e-5 by DIAMOND (Version 0.9.27). The taxon was generated into the kingdom, phylum, class, order, family, and genera levels. The ORF were assignment against the KEGG database using the DIAMOND (Version 0.9.27) with a cut-off E-value of 1e-5. For the alpha-diversity analysis, Shannon, simpson, Chao1, Abundance-based coverage estimator (ACE) index was calculated by vegan (Version 2.5-6) in R (Version 3.6.3). The Chao and ACE indices are used to measure community abundance. The larger the Chao and ACE, the higher the community richness. LEfSe analysis was used to identify taxa differences among groups, using the default parameters.

Breast milk was divided into tertiles as high, medium, or low according to the SCFA content, and the differences of intestinal microbiota in alpha diversity, composition and KEGG pathways were compared among the groups used Kruskal-Wallis test.

### Statistical analysis

Continuous variables are described statistically by mean ± standard deviation (SD) or median (P25, P75), and categorical variables are described by proportion or composition ratio. The Shapiro–Wilk method was used to investigate whether the distribution of data variables obeyed normality. Spearman's correlation was used to analyze the correlation between the content of SCFAs in breast milk and the microbiota in breast milk, and the correlation between microbiota in breast milk and infant intestinal microbiota. R 3.6.2 (R Development Core Team, Vienna, Austria) was used to calculate the strength of the symbiotic relationship between the microbiota, and Gephi was used for visualization. Significant differences between groups were analyzed by the KW rank sum test. Differences were considered statistically significant at *p* < 0.05.

## Results

### Basic participant characteristics

The data for 50 pairs of mothers and infants are shown in Table 1. The average post-natal age was 69.3 ± 40.5 days. Among them, 62.0% of mothers had given birth under the age of 30. The average birth weight of the infants was 3.4 ± 0.7 kg. Among the 50 nursing mothers, there were 26 in the normal weight range (BMI 18.5–23.9), accounting for 52%, and 24 in the overweight and obese group (BMI > 24.0). In addition, there were 9 people with a household monthly income (RMB: yuan) between 5,000 and 19,999, accounting for 18%; 29 people with a household monthly income between 20,000 and 49,999, accounting for 58%; and 12 people with an income >50,000.

**Table 1:** Basic characteristics of the 50 pairs of mother and infants [N(%) or Mean±SD].

**Characteristics**		**Description**
**Maternal age (years)**	18–30	31 (62.0)
	30–45	19 (38.0)
**Household monthly income (RMB: yuan)**		
	5,000–19,999	9 (18.0)
	20,000–49,999	29 (58.0)
	>50,000	12 (24.0)
**Post-natal days BMI (kg/m2)**	69.3 ± 40.5	
	18.5–23.9	26 (52.0)
	24.0–27.0	18 (36.0)
	≥27.0	6 (12.0)
**Infant birth weight (kg)**	3.4 ± 0.7	

### SCFAs in breast milk

We determined seven kinds of SCFAs in human milk with UPLC-MS, including C2, C3, C4, iC4, mC4, C5, and iC5, with an average content of 12.83 ± 36.67, 4.32 ± 2.43, 46.93 ± 38.86, 0.36 ± 3.29, 2.38 ± 1.59, 0.59 ± 0.49, and 0.98 ± 3.53 pmol/g, respectively, Table 2). The total SCFAs were 69.39 ± 86.83 pmol/g.

**Table 2:** Contents of short-chain fatty acids in breast milk.

**Fatty acids type**	**Fatty acids**	**Mean ±SD**	**Median IQR (25, 75)**
SCFAs (pmol/g)	C2	12.83 ± 36.67	4.83 (3.83, 5.67)
	C3	4.32 ± 2.43	3.92 (2.43, 5.81)
	C4	46.93 ± 38.86	34.88 (16.36, 69.10)
	iC4	1.36 ± 3.29	0.79 (0.57, 1.00)
	mC4	2.38 ± 1.59	1.93 (1.02, 3.41)
	iC5	0.98 ± 3.53	0.29 (0.19, 0.39)
	C5	0.59 ± 0.49	0.49 (0.19, 0.69)
	Total	69.39 ± 86.83	47.13 (24.59, 86.07)

### Composition of the breast milk microbiota

According to cluster tree analysis of the breast milk microbiota at the genus level (Supplementary Figure 1), the breast milk microbiota was mainly composed of four clusters, namely *Streptococcus* (27.19%), *Lactobacillus* (22.70%), *Staphylococcus* (20.38%), and *Enterococcus* (10.31%). The breast milk microbiota was mainly composed of *Lactobacillus gasseri, Enterococcus faecalis, Staphylococcus epidermidis*, and *Streptococcus salivarius* at the species level.

### Correlation between breast milk microbiota and infants' gut microbiota

First, we explored the correlation between the top 20 most-abundant breast milk microbiota and infant gut microbiota based on spearman analysis (Supplementary Figure 2), and found that there was a significant positive correlation between *Lactobacillus, Leuconostoc* and *Gardnerella* in breast milk and *Lactobacillus* in infant gut microbiota. *Enterococcus* and *Escherichia* in breast milk showed a significant positive correlation with *Enterobacter* in infant intestinal microbiota, while *Bifidobacterium* in breast milk showed a significant positive correlation with *Enterococcus* in infant intestinal microbiota.

### Correlation between breast milk SCFAs and the microbiota

**Figure 1:**
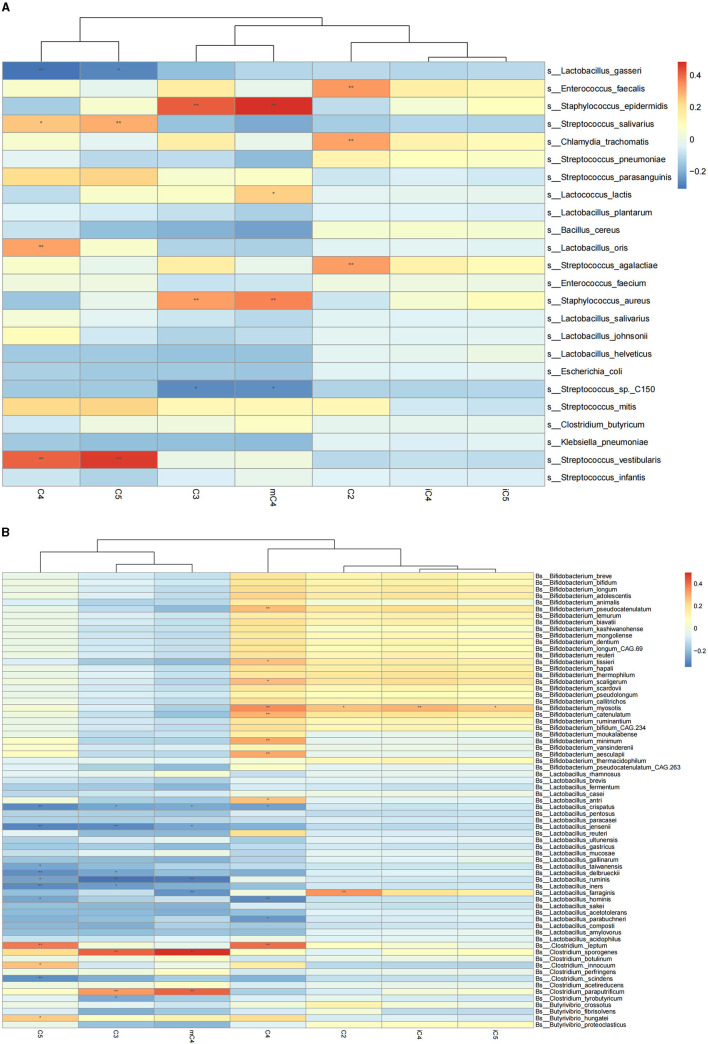
Correlation heatmap of breast milk SCFAs with the breast milk microbiota at the species level based on Spearman analysis. **(A)** Represents correlation heatmap of breast milk SCFAs with the top 25 most-abundant breast milk microbes at the species level. **(B)** Represents the correlation heatmap of SCFAs with Bifidobacterium, Lactobacillus, and Clostridium in breast milk. The horizontal axis represents the SCFAs detected in breast milk, and the vertical axis represents the microbiota at the species level in breast milk. The color represents the *r*-value of the correlation coefficient (unadjusted), red represents a positive correlation, blue represents a negative correlation, and the darker means a higher *r*-value, *p < 0.05, **p < 0.01.

We further explored the correlation of breast milk SCFA contents with the 25 most abundant breast milk microbes (Figure 1A). The results showed that the C2 content in breast milk was positively correlated with the abundance of *Streptococcus agalactiae* and *Enterococcus faecalis*. The contents of C3 and mC4 were negatively correlated with the abundance of *Streptococcus* spp. and positively correlated with that of *Staphylococcus epidermidis* and *Staphylococcus aureus*. Meanwhile, the C4 content in breast milk was also positively correlated with the abundance of *Lactobacillus oris* and negatively correlated with the abundance of *Lactobacillus gasseri*. The mC4 content of breast milk was positively correlated with the abundance of *Lactobacillus lactis*. Breastmilk C5 was also found to be correlated negatively with the abundance of *Lactobacillus gasseri* and positively with that of *Streptococcus salivarius* and *Streptococcus vestibularis*.

The correlations between SCFAs and the breast milk microbiota that have the potential ability to produce SCFAs were also examined, including *Bifidobacterium, Lactobacillus*, and *Clostridium* (Figure 1B). The results showed that the C2 content of breast milk was positively correlated with the abundance of *Bifidobacterium myosotis* and *Lactobacillus farraginis* in breast milk. The C3 content of breast milk was negatively correlated with the abundance of *Clostridium tyrobutyricum* in breast milk. The C4 content was correlated positively with the abundance of *Bifidobacterium pseudocatenulatum, Bifidobacterium tissieri, Bifidobacterium scaligerum, Bifidobacterium myosotis*, and *Lactobacillus antri* and negatively with that of *Lactobacillus crispatus* and *Lactobacillus hominis*. The contents of C3, mC4, and C5 were negatively correlated with the abundance of *Lactobacillus crispatus, Lactobacillus jensenii, Lactobacillus delbrueckii, Lactobacillus ruminis*, and *Lactobacillus iners* in breast milk. The abundances of C4 and C5 were positively correlated with that of *Clostridium leptum* in breast milk.

**Figure 2:**
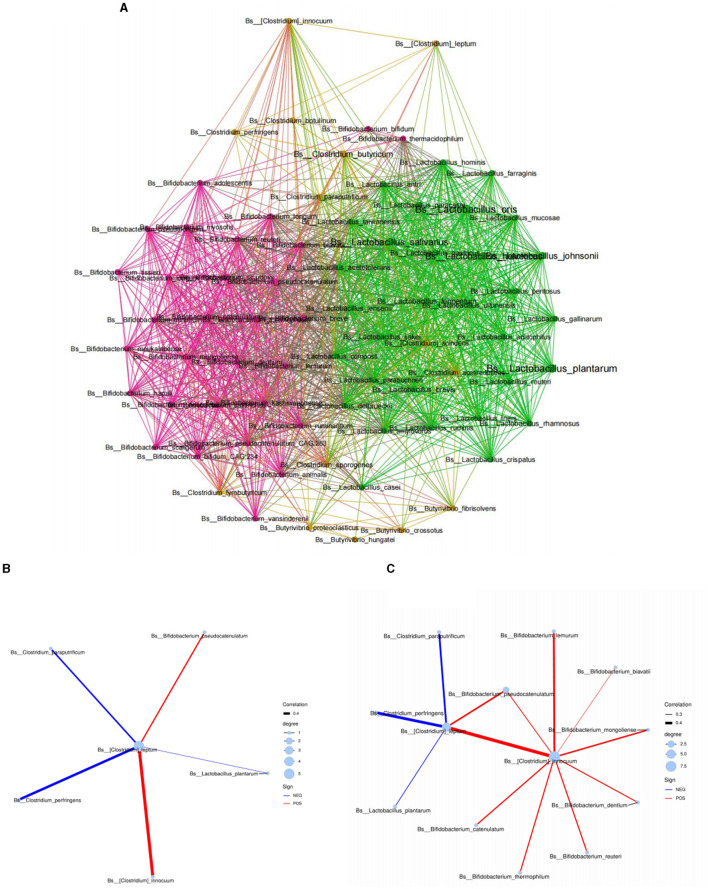
Symbiotic network diagram of breast milk microorganisms based on the Spearman relationship. **(A)** Symbiotic network diagram of Bifidobacterium, Lactobacillus, and Clostridium in breast milk. **(B, C)** Respectively represent the symbiotic relationship between Clostridium leptum in human milk and different Bifidobacterium spp. at the species level. Red represents a positive correlation, blue represents a negative correlation, and the thickness of the lines represents the strength of the correlation.

Based on the above results, we found that C4 was significantly related to the level of various *Bifidobacterium* spp. in human milk. We hypothesized that the main species of C4-producing bacteria may have an interactive symbiosis with specific *Bifidobacterium* spp. in human milk. Therefore, we further conducted the above microbiota analysis through a network symbiosis relationship diagram to explore its symbiosis inference. According to the analysis of the Spearman symbiotic relationship (Figure 2A), *Clostridium leptum* in breast milk had a direct symbiotic relationship with *Bifidobacterium pseudocatenulatum* and *Clostridium innocuum* in human milk (Figure 2B). Moreover, *Clostridium leptum* had an indirect symbiotic relationship with most *Bifidobacterium* spp. in breast milk, such as *Bifidobacterium reuteri, Bifidobacterium lemurum, Bifidobacterium biavatii, Bifidobacterium mongolinse*, and *Bifidobacterium dentium*, through *Clostridium innocuum* (Figure 2C), which suggest that the main species of C4-producing bacteria have an interactive symbiosis with specific *Bifidobacterium* spp. in human milk.

### Association of breast milk SCFAs with infants' intestinal microbiota

We categorized the SCFA levels in breast milk into high, medium, and low according to their content. Then, we further analyzed the differences in the diversity and composition of infants' intestinal microbiota among different breast milk SCFA groups. For the alpha diversity index, there were no significant differences between the C2, C4, iC4, mC4, C5, and iC5 groups in the Chao, ACE, or Shannon and Simpson indexes (Supplementary Figure 3). The Chao and ACE indexes of the high C3 group were significantly higher than those of the low and medium groups.

PLS-DA analysis showed that the contents of different SCFAs in breast milk, including C2, C3, C4, iC4, mC4, C5, and iC5, were significantly associated with the overall composition of the infant intestinal microbiota (Supplementary Figure 4).

To further analyze the impact of milk SCFAs on the specific composition of the infant intestinal microbiota, we analyzed the above association at the genus and species levels, respectively (Supplementary Figure 5). The results showed that the infant intestinal microbiota was mainly composed of *Bifidobacteria, Escherichia, Lactobacillus*, and *Bacteroides* at the genus level, and the content of SCFAs in human milk was mainly related to the composition of *Bifidobacterium, Lactobacillus*, and *Lacticaseibacillus* in infants' microbiota.

**Figure 3:**
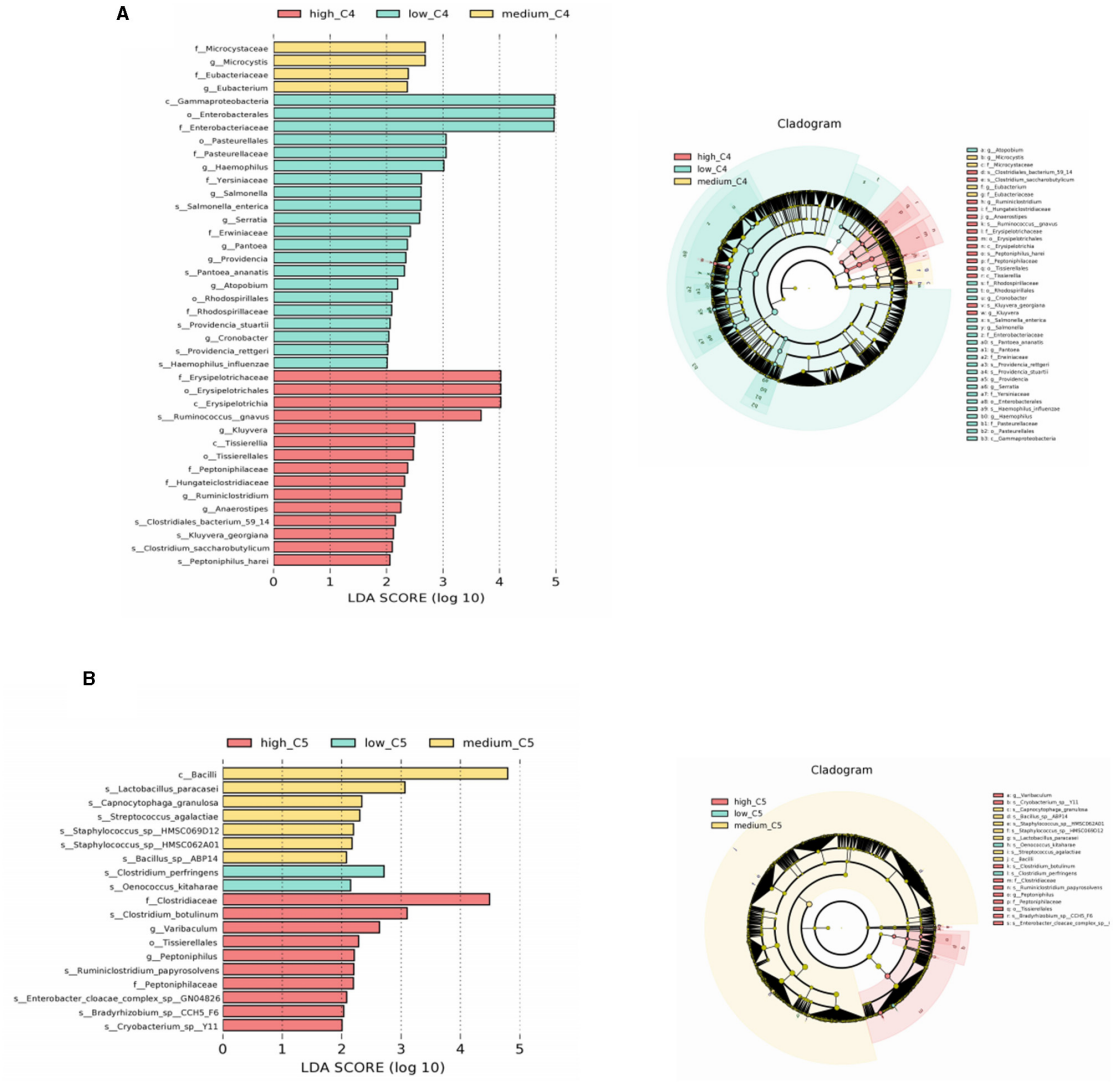
Biomarkers of differences between different SCFA content groups in human milk. **(A, B)** Represent LEfSe analysis of C4 and C5 groups, respectively. Taxonomic cladogram obtained from LEfSe and linear discriminant analysis (LDA) of these groups. Biomarker taxa are highlighted with colored circles and shaded areas. Each circle's diameter reflects the abundance of those taxa in the community, using a cutoff value of ≥2.0. C4: low (6.48–20.57 pmol/g), medium (21.81–45.11 pmol/g) and high content (47.05–140.11 pmol/g); C5: low (0.10–0.29 pmol/g), medium (0.33–0.63 pmol/g) and high content (0.64–2.35 pmol/g).

LEfSe analyses of the infants' intestinal microbiota were conducted to determine significant biomarkers of different breast milk SCFA levels. It was found that infants in the low C4 tertile (6.48–20.57 pmol/g) had a relatively high abundance of *Salmonella* and *Salmonella enterica*; the medium C4 tertile (21.81–45.11 pmol/g) had a relatively high abundance of *Microcystis* and *Eubacterium*, and the high C4 tertile (47.05–140.11 pmol/g) had a relatively high abundance of *Ruminococcus gnavus* and *Clostridiales bacterium* (Figure 3A). A significantly different microbial biomarker was also found for C5, in which the medium tertile had a relatively high abundance of *Lactobacillus paracasei, Streptococcus agalactiae*, and *Staphylococcus* sp. and the high C5 tertile (0.64- 2.35 pmol/g) had a relatively high abundance of *Clostridiaceae* and *Ruminiclostridium papyrosolvens* (Figure 3B). Biomarkers of other SCFAs were not significantly different, and the results are not shown.

### Functional pathway

Based on the above analysis of significant biomarkers of the intestinal microbiota at the species level, we screened the key SCFAs (C4 and C5) in breast milk for their effects on metabolic pathways by conducting a functional correlation study. We classified the metabolic pathways at the functional level using KEGG pathway analysis. The 50 infants' microbiota corresponded to 6 first-level, 45 second-level, and 422 third-level metabolic pathways. Focusing on the studied population, we attempted to analyze 200 out of the 422 tertiary metabolic pathways under 17 KEGG secondary metabolic pathway branches (including carbohydrate metabolism, amino acid metabolism, cofactors and vitamins, lipid metabolism, circulatory system, digestive system, endocrine system, excretory system, immune system, etc.), which are strongly related to early health, growth, and development.

**Figure 4:**
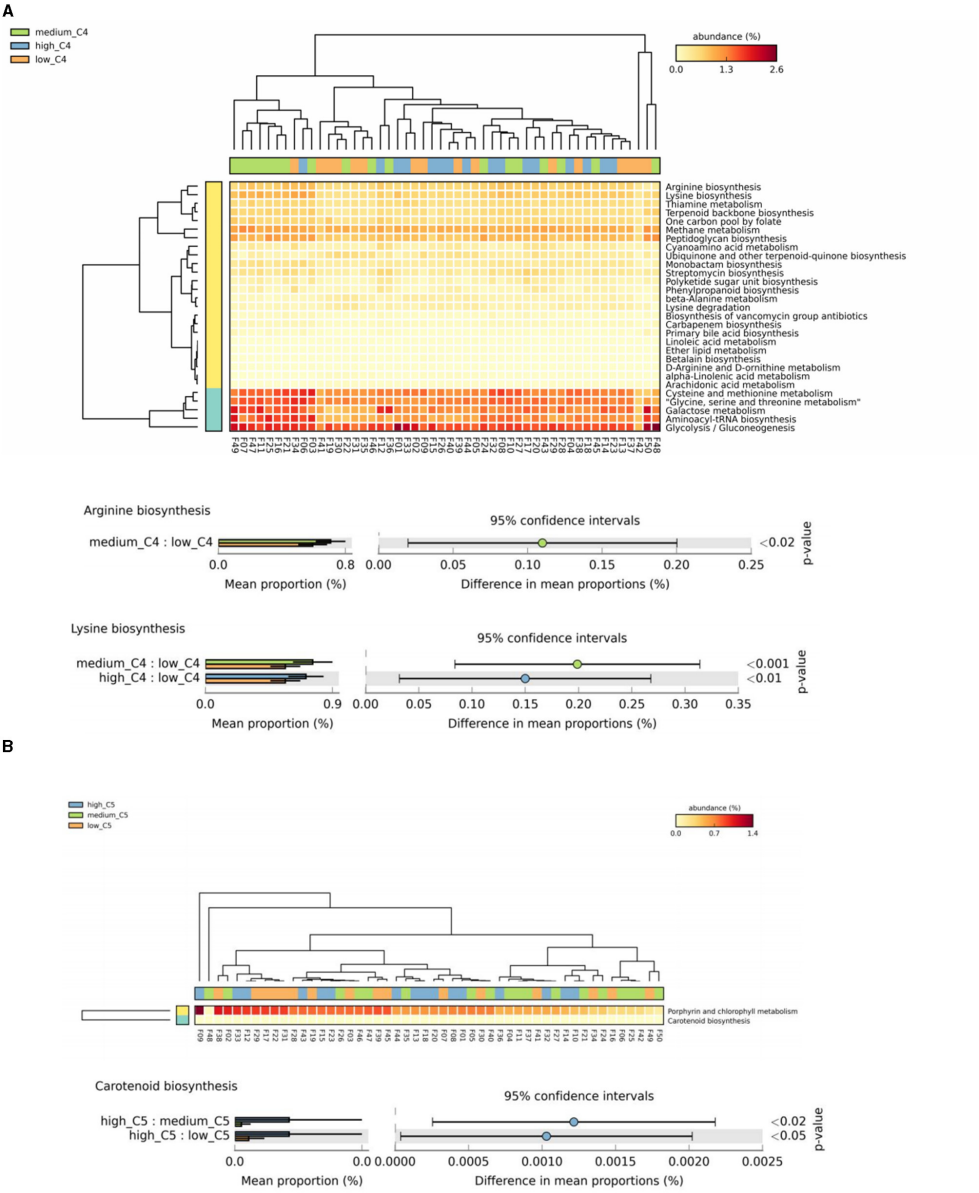
Analysis of specific SCFAs and KEGG tertiary metabolic pathway abundance levels in human milk with different SCFA contents. **(A, B)** Represent the KEGG tertiary enrichment and differential pathways of breast milk C4 and C5, respectively, in different content groups (orange, green, and blue indicate low, medium, and high content, respectively). A Kruskal–Wallis rank sum test was used to compare the KEGG functional levels of different SCFA groups in breast milk (orange, green, and blue indicate low, medium, and high content, respectively). The horizontal coordinate represents the sample information detected. Yellow to red indicates the abundance distribution of different samples in tertiary metabolic pathways from low to high. The lower bar represents the tertiary metabolic pathways with significant differences. Differences were considered significant at *p* < 0.05. C4: low (6.48–20.57 pmol/g), medium (21.81–45.11 pmol/g) and high content (47.05–140.11 pmol/g); C5: low (0.10–0.29 pmol/g), medium (0.33–0.63 pmol/g) and high content (0.64–2.35 pmol/g).

The results showed significant differences in arginine and lysine biosynthesis among different breast milk C4 levels, in which the high and medium groups had a higher abundance than the low C4 content groups (Figure 4A). There were also significant differences in carotenoid biosynthesis among C5 groups, in which the high tertile had a relatively high abundance (Figure 4B).

**Figure 5:**
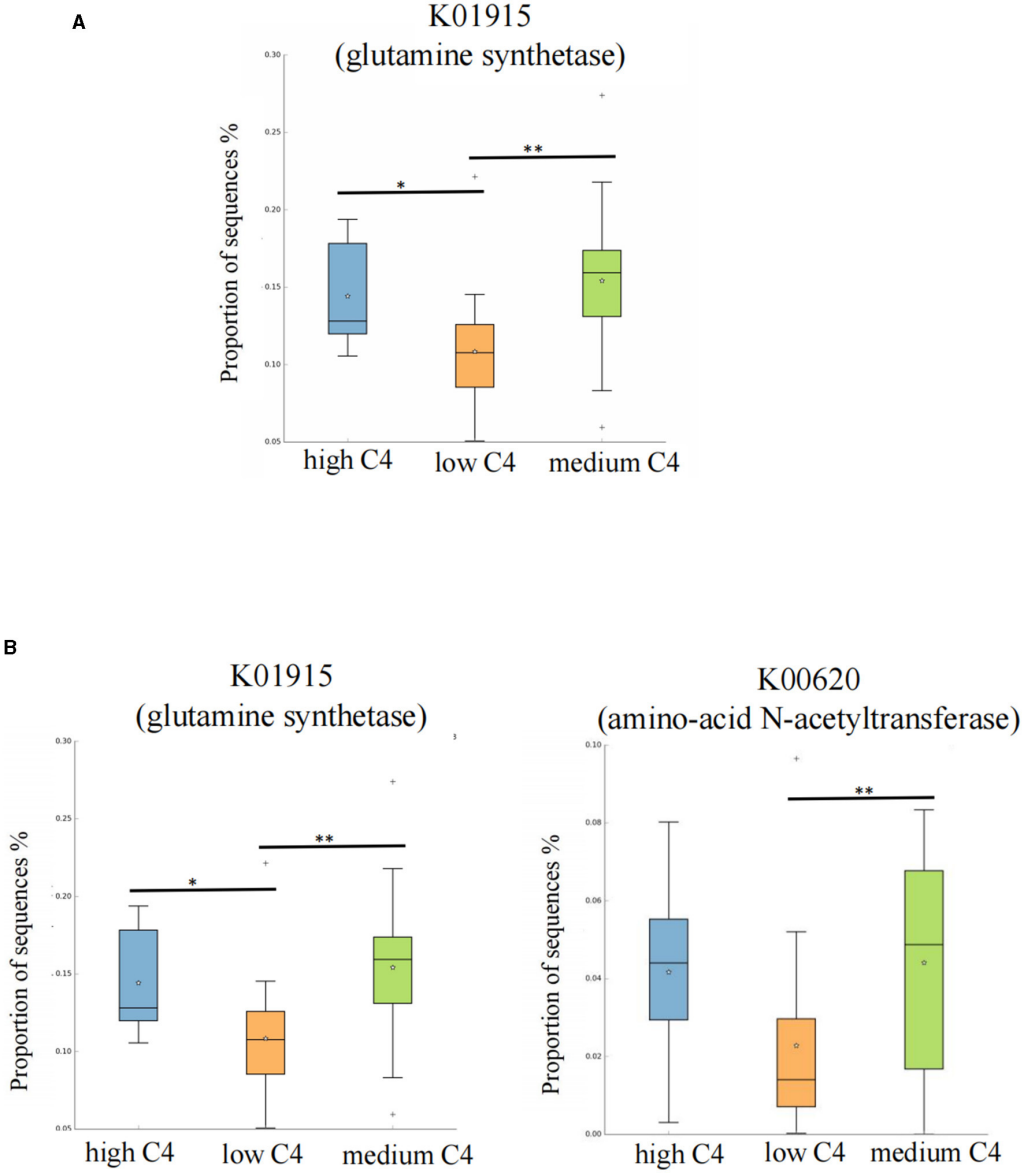
Kyoto Encyclopedia of Genes and Genomes Orthology (KO) analysis of significant differences for certain breast milk SCFAs. **(A, B)** Represent the KO analysis of breast milk C4 of the arginine and lysine biosynthesis pathways, and the results are shown in the box diagram (orange, green, and blue indicate low, medium, and high content, respectively). The horizontal coordinate represents the different SCFAs groups, and the vertical coordinate represents the abundance of the corresponding differential KO, in which K01915 corresponds to glutamine synthetase [EC:6.3.1.2], and K00620 corresponds to amino-acid N-acetyltransferase [EC:2.3.1.35 2.3.1.1]. *p < 0.05. C4: low (6.48–20.57 pmol/g), medium (21.81–45.11 pmol/g) and high content (47.05–140.11 pmol/g); C5: low (0.10–0.29 pmol/g), medium (0.33–0.63 pmol/g) and high content (0.64–2.35 pmol/g). **p < 0.01.

We further searched the KO (Kyoto Encyclopedia of Genes and Genomes Orthology) corresponding to the above-mentioned differential metabolic pathways in the KEGG database. For C4, the most significant corresponding signal pathways were arginine biosynthesis and lysine biosynthesis, which involved 61 and 112 related KO reported in the KO database, respectively. When these KO were compared to the total of 10,016 KO annotated with the microbiota data from this study, it was found that 49 KO were under the arginine biosynthesis pathway, and 88 were under the lysine biosynthesis pathway. A KW rank sum test was used to compare the relative KO abundances between C4 content groups. The functional relative abundances of K01915 (in the arginine biosynthesis pathway), which corresponds to glutamine synthetase [EC:6.3.1.2], were significantly higher in the medium and high C4 groups (Figure 5A). K00620 (in lysine biosynthesis), which corresponds to amino acid N-acetyltransferase [EC:2.3.1.35 2.3.1.1], was significantly higher in the medium and high C4 group. K01915 (in lysine biosynthesis), which corresponds to glutamine synthetase [EC:6.3.1.2], was significantly higher in the medium and high C4 groups (Figure 5B). There was no significant difference in the KO involved in different signal pathways between C5 content groups.

## Discussion

In this study, the associations between the breast milk microbiota and SCFAs in milk were investigated by correlation analysis, and the potential effect of different contents of breast milk SCFAs on infants' intestinal microbiota were further explored. To the best of our knowledge, we are the first to report that the specific *Bifidobacterium* spp. in human milk may have an interactive symbiosis with main species of C4-producing bacteria and help to produce C4 in breast milk. We also suggested that certain breast milk SCFAs that might be involved in inhibiting infant gut pathogens, such as C4, were found to be significantly associated with lower prominence of certain microbial species in the infants' gut, including *Salmonella, Salmonella enterica*, and *Eubacterium*. Moreover, based on KEGG pathway analysis, the changes of microbiota associated with SCFAs related to the key metabolic pathways for infants.

### SCFAs in breast milk and their relationship with the breast milk microbiota

Current research demonstrates that SCFAs in breast milk may be produced by the mother's gut bacteria and transported to the mammary gland through the circulatory system (Stinson et al., 2020; Stinson and Geddes, 2022). Scientists also theoretically suggested that milk SCFAs could be metabolized by bacteria in breast milk (Stinson and Geddes, 2022); however, there is no experimental evidence to support this hypothesis, and the real-world data on the association between SCFAs and the microbiota in human breast milk are limited. Based on the current results, we did not find significant associations of C2, C3, C5, and iC5 in breast milk with *Bifidobacterium* and *Lactobacillus* in breast milk, which were considered as the main SCFA-producing microbials (Yang et al., 2022). The above results indirectly supports the idea that these milk SCFAs came from the maternal gut.

Surprisingly, we also noticed that the levels of C4 and C5 in breast milk were significantly positively correlated with the abundance of *Clostridium leptum* in breast milk. *Clostridium leptum* is the most abundant butyrate-producing bacteria. Moreover, according to the symbiotic network relationship analysis, breast milk C4-producing bacteria, such as *Clostridium leptum* and *Clostridium innocuum*, were observed to be significantly correlated with *Bifidobacterium* at the species level in breast milk. This phenomenon was also observed in *in vitro* experiments and the human gut (Prentice et al., 2019; Stinson et al., 2020). It could be explained by the fact that most butyrate-producing bacteria cannot directly ferment prebiotics, while some lactic acid bacteria (e.g., *Bifidobacterium*) can partially hydrolyze prebiotics that are difficult to digest and produce intermediate metabolites, such as short oligosaccharides and monosaccharides. In addition, lactic acid bacteria can also completely hydrolyze certain prebiotics to produce metabolic end products, such as C2 and lactic acid, and then allow the butyrate-producing bacteria to further ferment these products as secondary substrates to produce C4. Therefore, although the symbiotic network was first observed in human breast milk, we could reasonably speculate that breast milk C4 could be produced by C4-producing bacteria in breast milk with the help of lactic acid-producing bacteria.

### Association of breast milk SCFAs and infant intestinal microbiota

This study further revealed that the composition of the intestinal microbiota in infants is significantly associated with maternal SCFAs. According to the results of diversity analysis, we observed several SCFAs associated with the overall composition and distribution of infants' intestinal microbiota.

SCFAs induce a wide range of biological effects through interactions with G protein-coupled receptors and inhibition of histone deacetylase, including antibacterial and anti-inflammatory activities and promoting the integrity of the intestinal epithelial barrier (Chen et al., 2023). However, most previous findings based on *in vitro* studies or investigated on SCFAs exhibited in the gut rarely reported SCFA in breast milk and their functions. Our study extended the previous findings by illustrating that the breast milk SCFAs could also perform certain functions in infants. We observed significant differences in gut microbial composition between different breast milk SCFA levels according to LEfSe analysis. The results showed that the content of *Salmonella* in the low C4 group was relatively high, while that of *Eubacterium* and *Clostridiales bacterium* in the medium and high C4 groups were relatively high, which might indicate that consuming breast milk with a high C4 could help to inhibit the growth of intestinal pathogenic bacteria. A previous animal study, which showed that butyrate could inhibit the spread of *Salmonella* in the intestine and further enhance the barrier function of the gut mucosa, supported our findings (Homann et al., 2023).

### Function analysis based on KEGG pathway analysis

Considering the potential function of the abovementioned different infant intestinal microbes regulated by SCFAs, we further conducted KEGG pathway analysis to explore the potential gene expression differences and metabolic pathways among different C4 levels. The results revealed that C4 is associated with high expression of K00620 in the lysine biosynthesis pathway, corresponding to amino-acid N-acetyltransferase [EC:2.3.1.35 2.3.1.1]. Amino acid acetyltransferase can transfer the acetyl group in acetyl-CoA to the amino group in the amino acid molecule and is a key enzyme in lysine bio-anabolism (Liu et al., 2022; Ghasemi et al., 2023). As an essential amino acid for infants, lysine plays an important role in regulating the physiological activity of intestinal epithelial cells and inhibiting the colonization of harmful bacteria (Wang et al., 2023). Gebeyew et al. (2021) reported that dietary lysine supplementation improved intestinal barrier function and down-regulated mRNA expression of intestinal inflammatory factors such as IL-1β, TNF-α, interferon-γ, and toll-like receptor-4. Lysine-derived Maillard reaction products inhibit the growth of Salmonella enterica (Wong et al., 2023). The mechanism of their inhibition of Salmonella may be closely related to lysine acetylation, which can inhibit protein activity and DNA binding ability in pathogenic bacteria (Koo et al., 2020). Meanwhile, C4 is closely related to modification by acetylation (Kang et al., 2023).

Similarly, we also identified that K01915 [glutamine synthetase (EC:6.3.1.2)] was highly expressed in medium and high C4 breast milk groups, which response to the arginine biosynthesis metabolism (Xie et al., 2023). Arginine plays an important role in regulating intestinal epithelial cells and inhibiting the expression of inflammatory factors (Ge et al., 2022). At present, the relationship between the C4 content in breast milk and arginine metabolism has not been reported. Although our study revealed that C4 levels in breast milk may be related to infant arginine metabolism, this finding should be validated in future studies. In summary, according to KEGG analysis, we can further demonstrate that breast milk C4 may inhibit the abundance of harmful bacteria by regulating amino acid metabolism and intestinal mucosal barrier function.

### Strengths and limitations

The strength of this study lies in its use of metagenome sequencing, which can identify specific intestinal bacteria at the species level that could be affected by SCFAs. We also adopted the method of maternal–infant pairs to explore the role of breast milk in infant health. Combined with KEGG analysis, the data could further clarify the function of breast milk SCFAs and provide a more detailed explanation for the mechanisms involved in their function.

Four limitations should be addressed in our study. Firstly, as it was a pilot study, the sample size was limited. Secondly, this study was a cross-sectional study, and causation cannot be fully determined. Thirdly, although several confounders were taken into consideration in this study, there are plenty confounding factors in breast milk that could affect the results, such as other fatty acid and prebiotic-like ingredients. Future prospective studies with a large sample size are required to confirm the results of this study and further examine the long-term effects of breast milk SCFAs on infant's intestinal microbiota and development. Fourthly, in this study, SCFA contents in infant feces were not detected due to the limited amount of infant stool samples collected. Account the SCFA contents analysis in future study could provide additional insights into how SCFAs from breast milk influence the gut microbiota of infants.

## Conclusion

Based on whole genome shotgun sequencing and UPLC-MS analyses, the current results suggest that in human milk the specific *Bifidobacterium* may have an interactive symbiosis with main species of C4-producing bacteria and help to produce C4 in breast milk. Certain breast milk SCFAs, such as C4, might be involved in inhibiting infant gut pathogens. The current study reveals that C4 might correlate with increased abundance or presence of specific enzymes, which corresponds to the potential regulatory function of amino acid metabolism. These findings may illustrate the potential bond between maternal and infant health via breast milk SCFAs. In the future, large-scale prospective studies can be conducted to further explore the long-term effects and mechanisms of breast milk SCFAs on the gut microbiota and development of early life.

## Data availability statement

The datasets presented in this study can be found in online repositories. The names of the repository/repositories and accession number(s) can be found in the article/Supplementary material.

## Ethics statement

The protocol was approved by the Research Center for Public Health, of Tsinghua University (No. THUSM/PHREC/2021-003). The studies were conducted in accordance with the local legislation and institutional requirements. Written informed consent for participation in this study was provided by the participants' legal guardians/next of kin.

## Author contributions

MX: Conceptualization, Data curation, Formal analysis, Investigation, Methodology, Writing – original draft. YY: Data curation, Formal analysis, Investigation, Writing – original draft. SD: Data curation, Formal analysis, Investigation, Writing – original draft. TL: Data curation, Formal analysis, Investigation, Writing – original draft. IS: Data curation, Formal analysis, Investigation, Writing – original draft. AZ: Project administration, Supervision, Writing – review & editing.
