# Supplementary material for: Short-chain fatty acids in breast milk and their relationship with the infant gut microbiota
Source: Front Microbiol. 2024 Feb 19;15:1356462. doi: 10.3389/fmicb.2024.1356462 (PMC10909814; doi:10.3389/fmicb.2024.1356462)
Supplement: Supplementary file 1 [file Data_Sheet_1.PDF]

genus

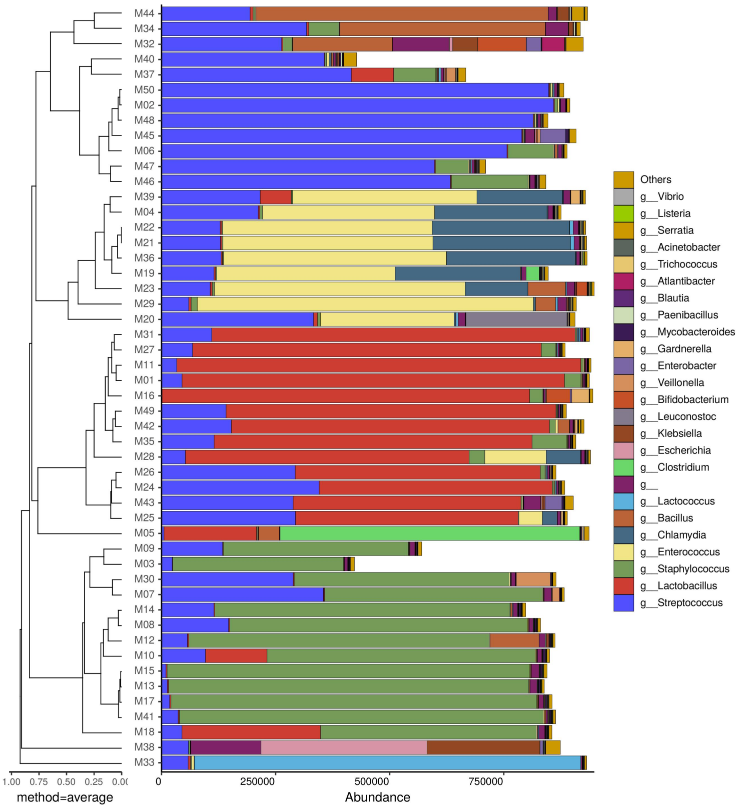

species

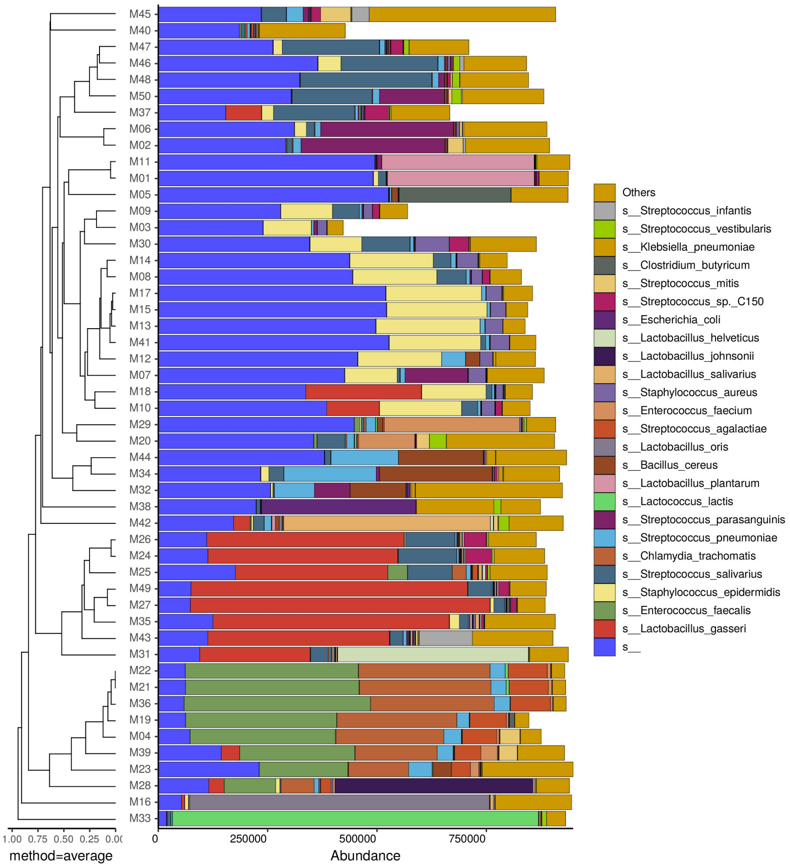

Supplementary Figure S1. The cluster tree analysis of the breast milk microbiota

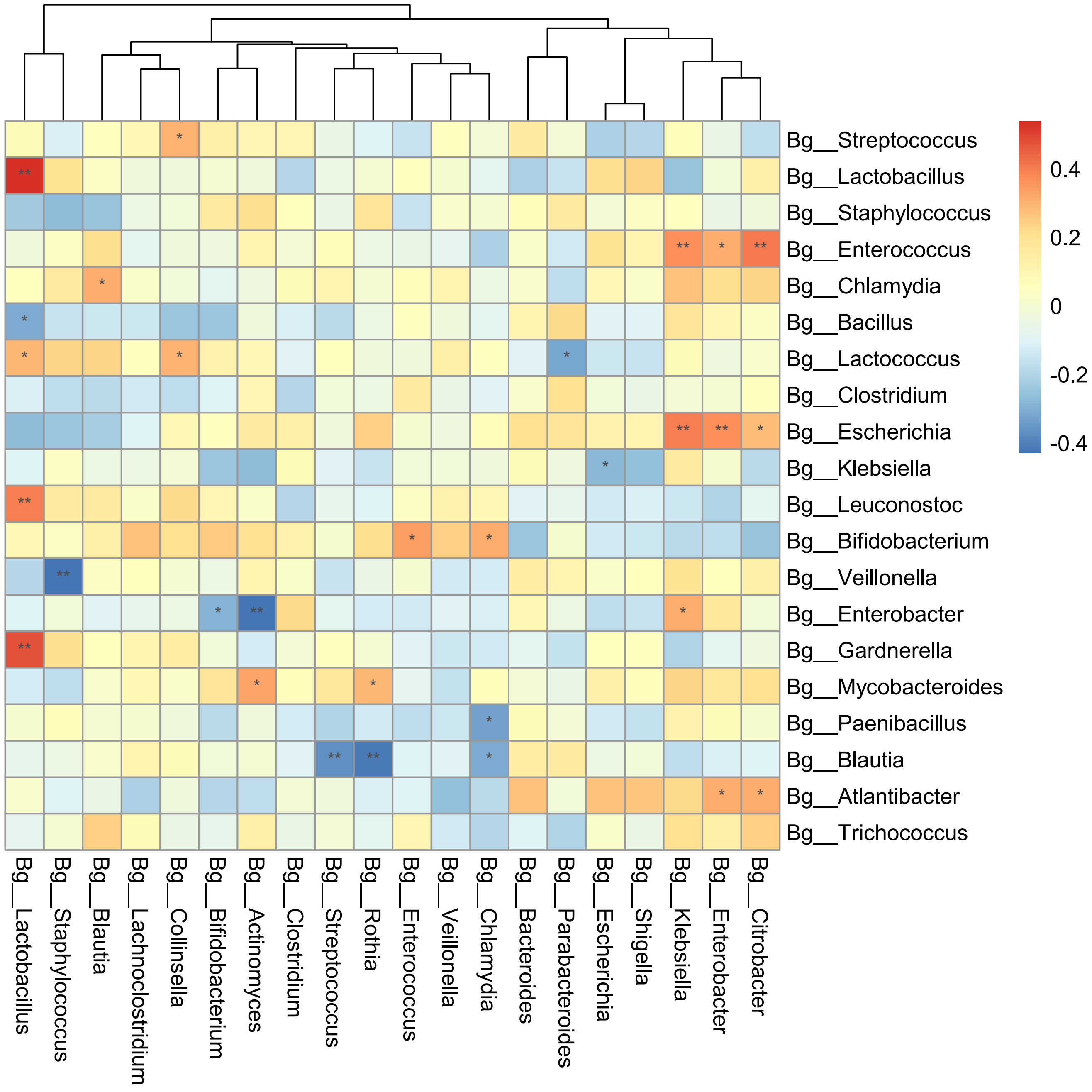

Supplementary Figure S2. Heatmap of correlation between infant gut microbiota and breast milk gut microbiota based on Spearman analysis. The horizontal axis represents the infant gut microbiota, and the vertical axis represents the breast milk microbiota

C2:0

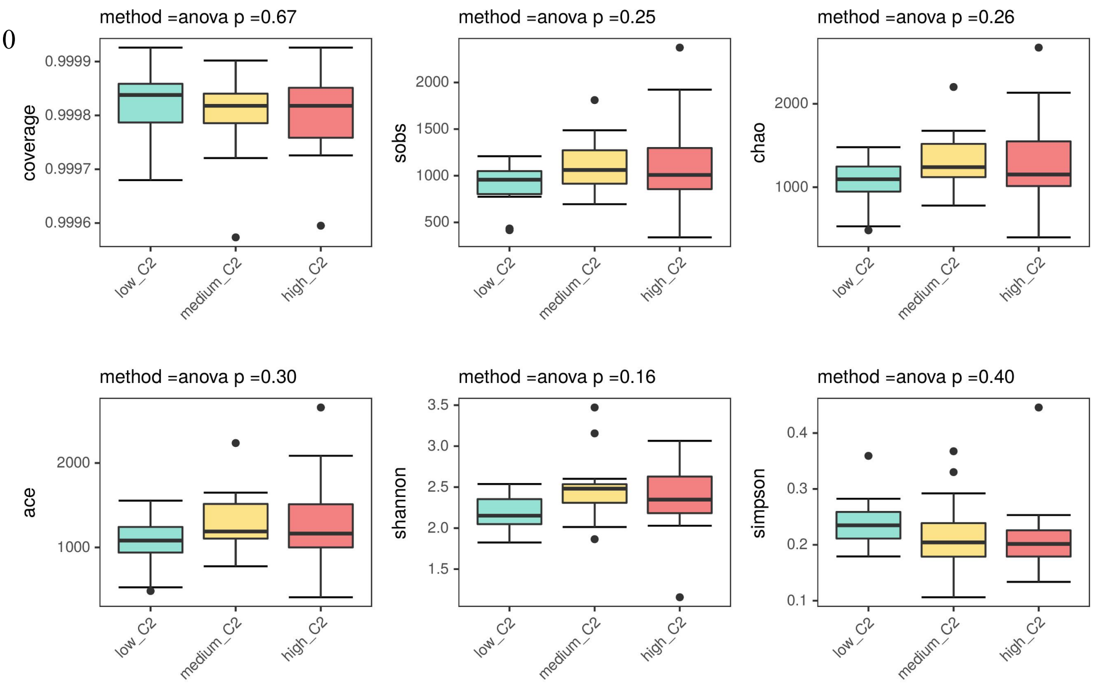
method =anova p =0.67
method =anova p =0.25
method =anova p =0.26
method =anova p =0.30
method =anova p =0.16
method =anova p =0.40
coverage
sobs
chao
ace
shannon
simpson
low_C2
medium_C2
high_C2

C3:0

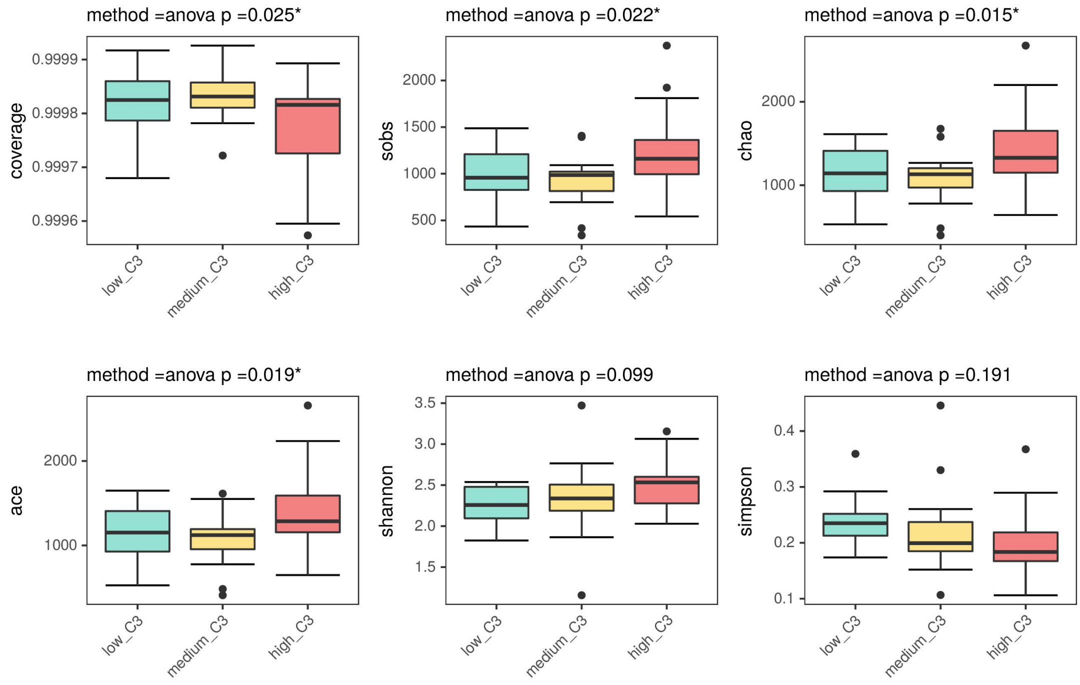
method =anova p =0.025*
method =anova p =0.022*
method =anova p =0.015*
method =anova p =0.019*
method =anova p =0.099
method =anova p =0.191
coverage
sobs
chao
ace
shannon
simpson
low_C3
medium_C3
high_C3

C4:0

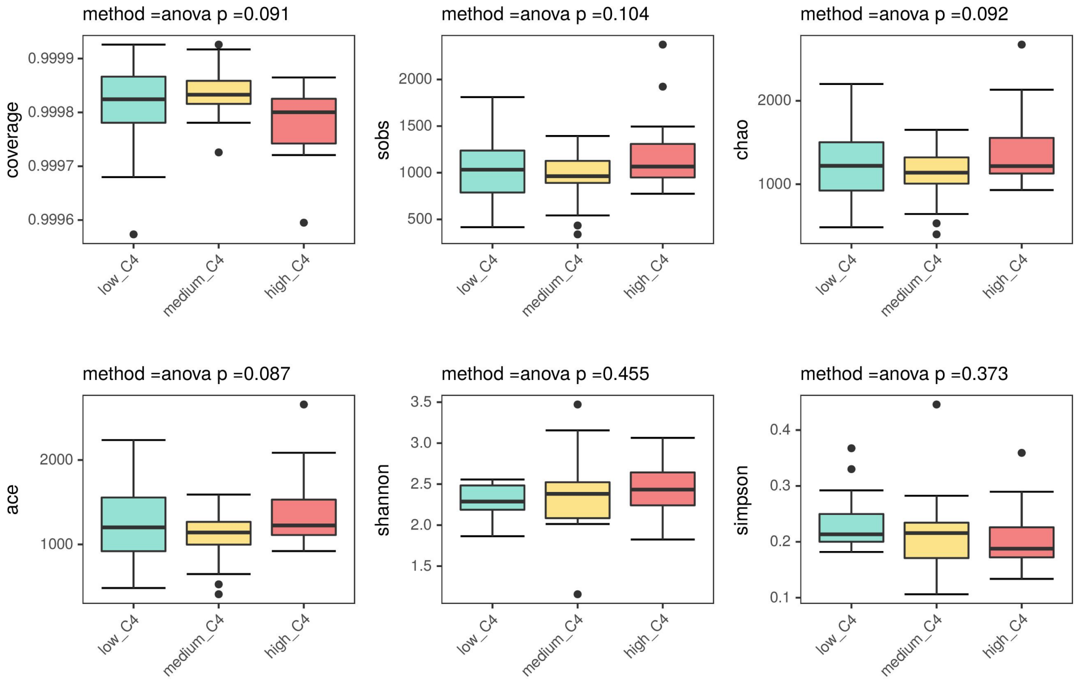
method =anova p =0.091
coverage
0.9999
0.9998
0.9997
0.9996
method =anova p =0.104
sobs
2000
1500
1000
500
method =anova p =0.092
chao
2000
1000
low_C4
medium_C4
high_C4
method =anova p =0.087
ace
2000
1000
method =anova p =0.455
shannon
3.5
3.0
2.5
2.0
1.5
method =anova p =0.373
simpson
0.4
0.3
0.2
0.1

iC4:0

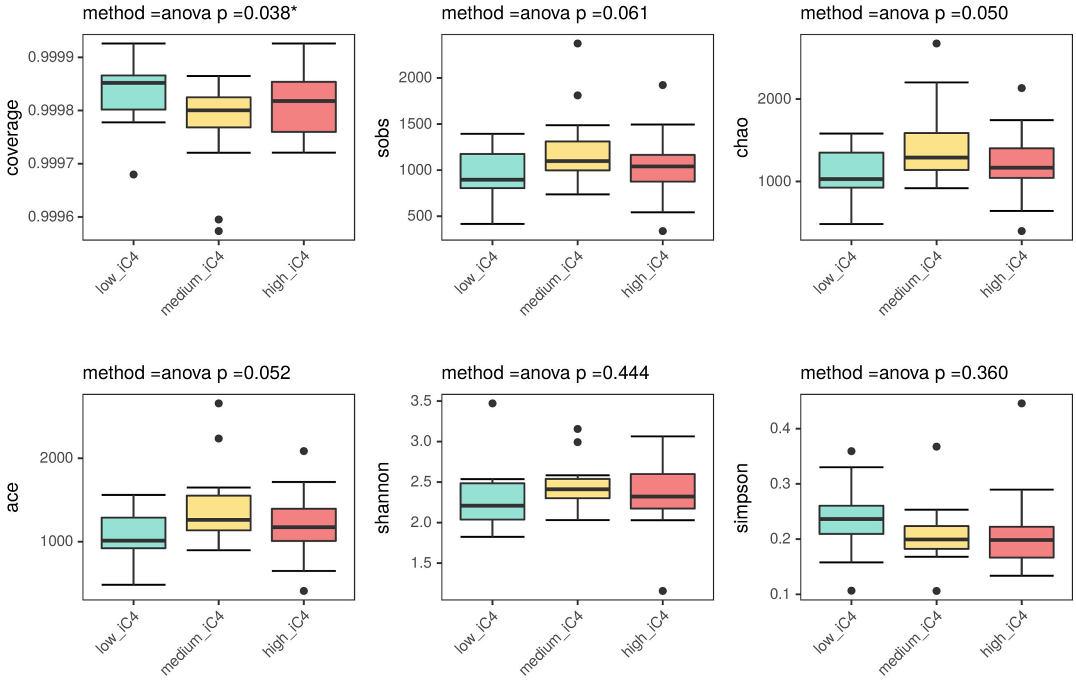
method =anova p =0.038*
coverage
0.9999
0.9998
0.9997
0.9996
method =anova p =0.061
sobs
2000
1500
1000
500
method =anova p =0.050
chao
2000
1000
low_iC4
medium_iC4
high_iC4
method =anova p =0.052
ace
2000
1000
method =anova p =0.444
shannon
3.5
3.0
2.5
2.0
1.5
method =anova p =0.360
simpson
0.4
0.3
0.2
0.1

mC4:0

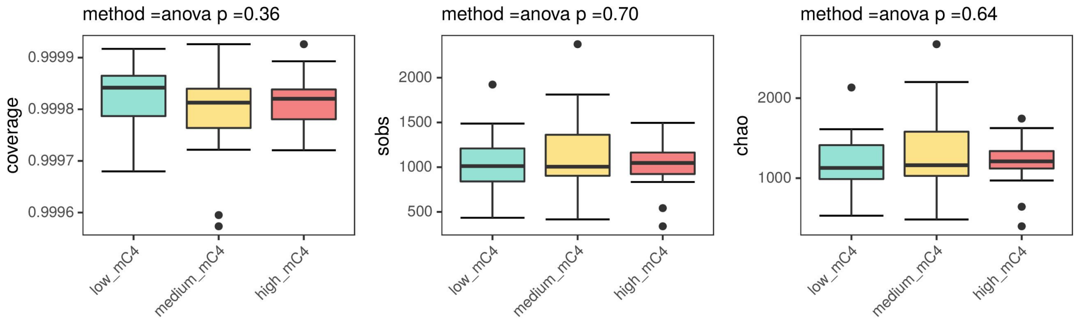

method =anova p =0.36
method =anova p =0.70
method =anova p =0.64
method =anova p =0.63
method =anova p =0.98
method =anova p =0.96
coverage
sobs
chao
ace
shannon
simpson
low_mC4
medium_mC4
high_mC4

iC5:0

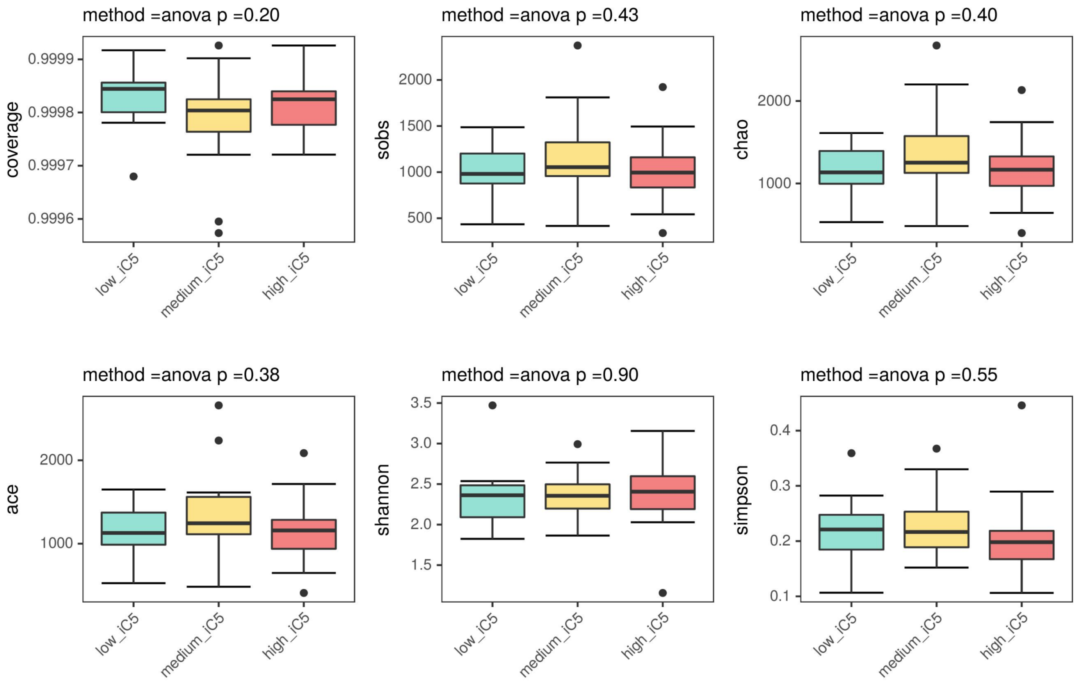

method =anova p =0.20
method =anova p =0.43
method =anova p =0.40
method =anova p =0.38
method =anova p =0.90
method =anova p =0.55
coverage
sobs
chao
ace
shannon
simpson
low_iC5
medium_iC5
high_iC5

C5:0

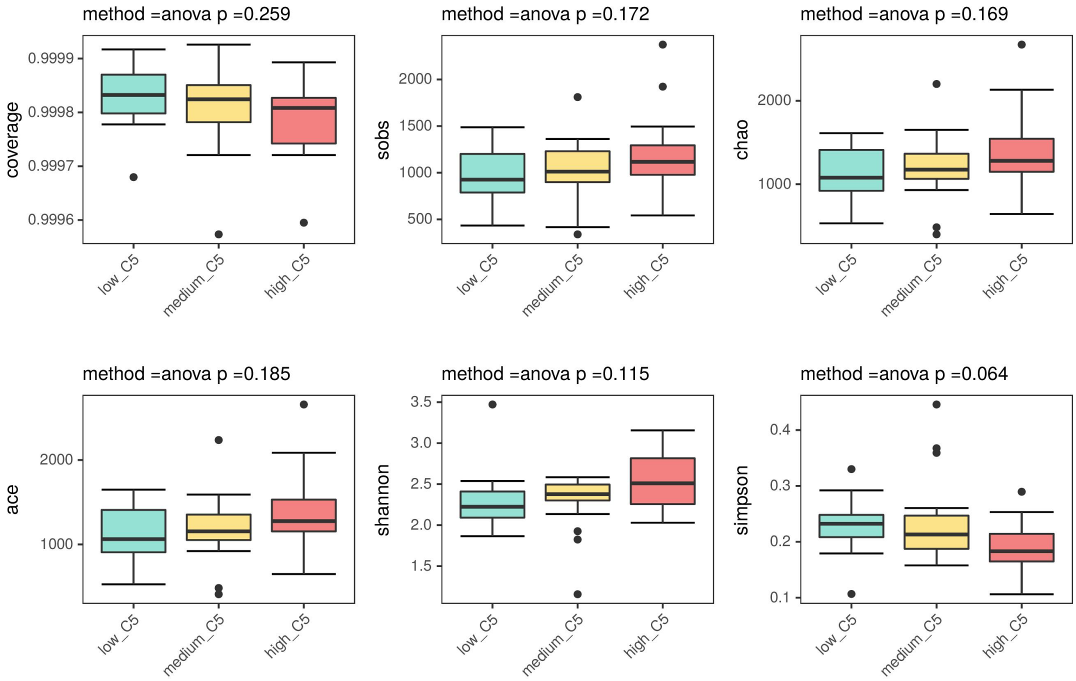

Supplementary Figure S3. The Chao1 index, ACE, and Shannon and Simpson indexes for the SCFAs in different groups of breast milk

C2:0

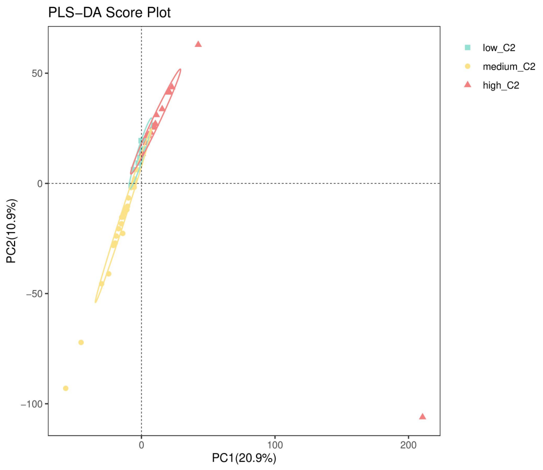

C3:0

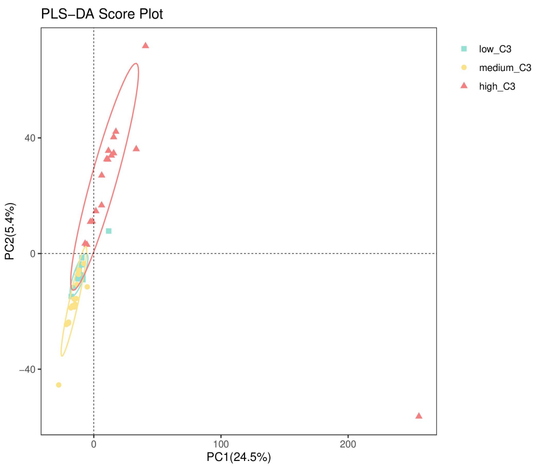

C4:0

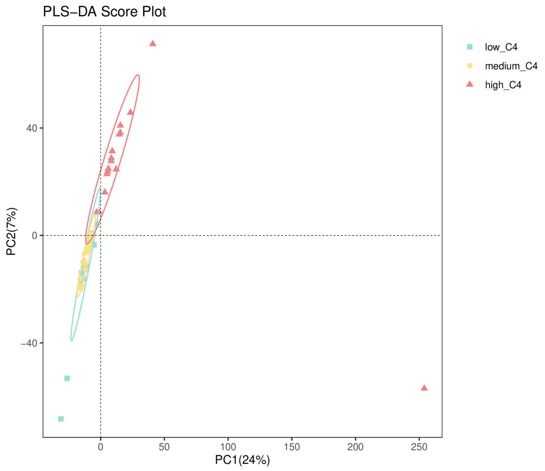

iC4:0

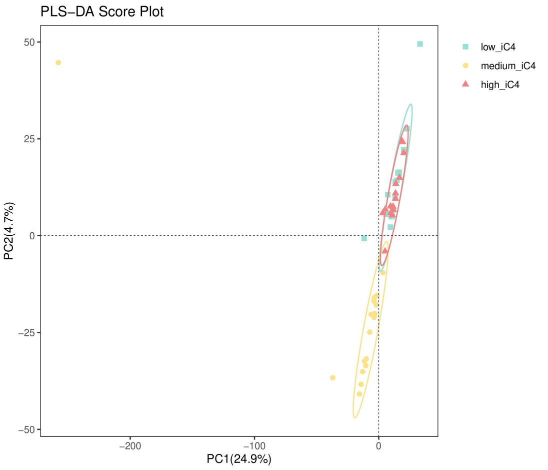

mC4:0

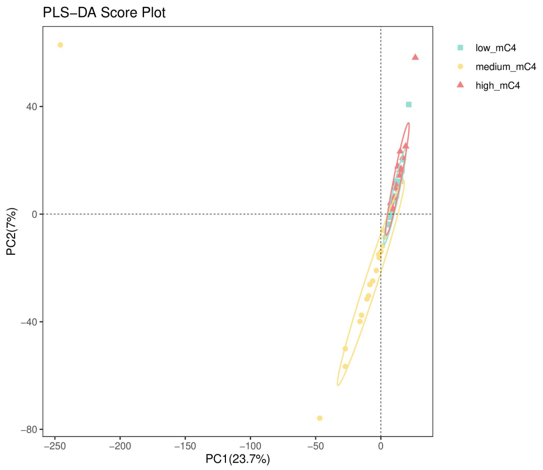

iC5:0

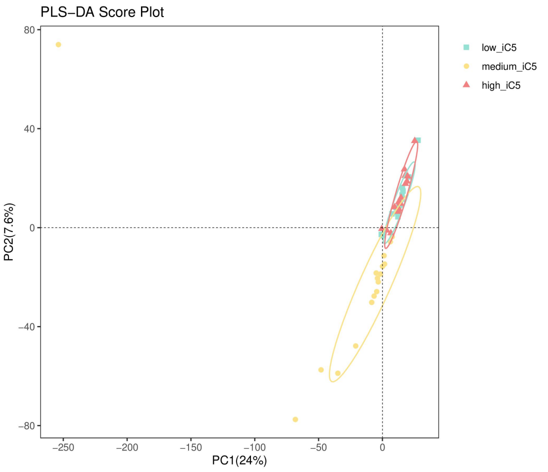

C5:0

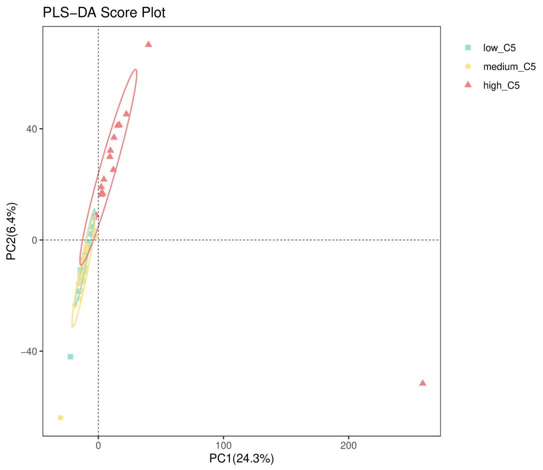

Supplementary Figure S4. The PLS-DA analysis for the SCFAs in different groups of breast milk.

SCFAs genus species

C5

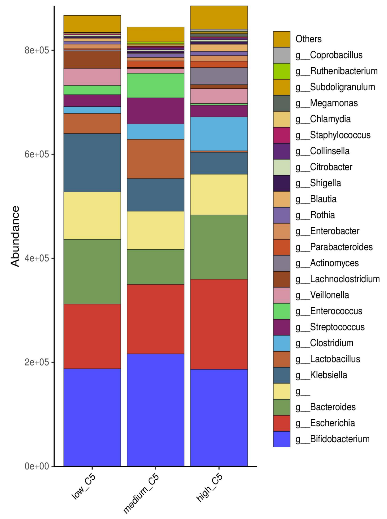

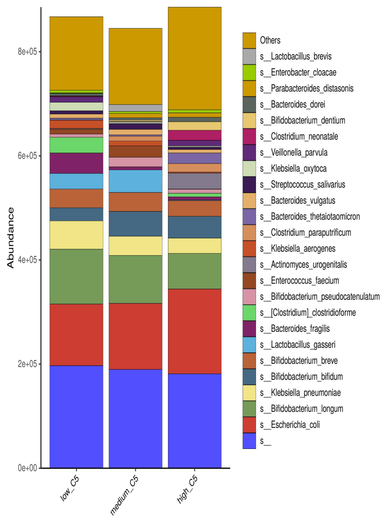

iC5

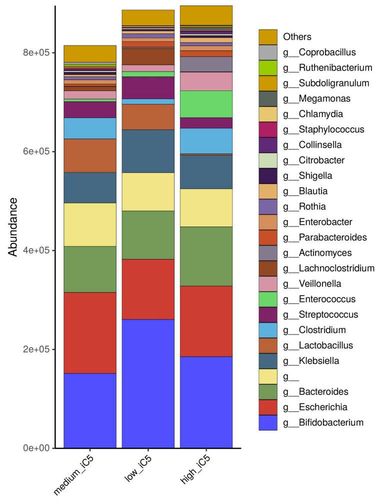

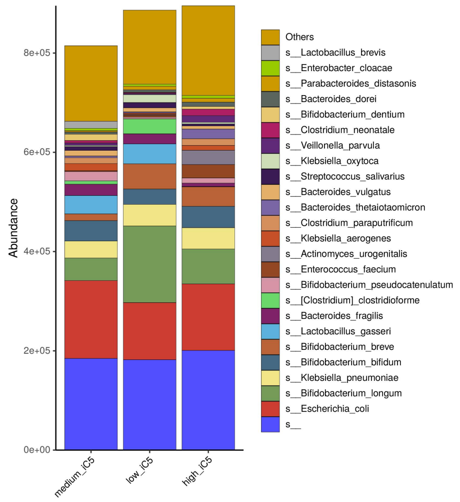

iC4

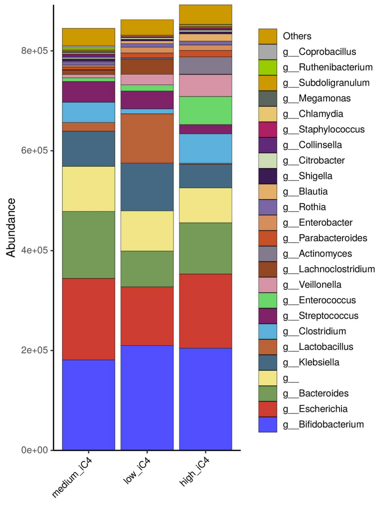

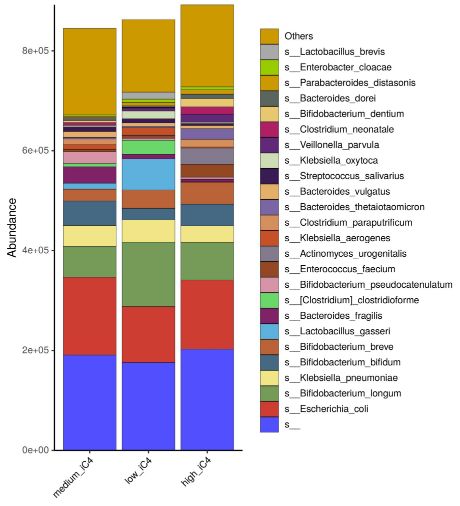

C4

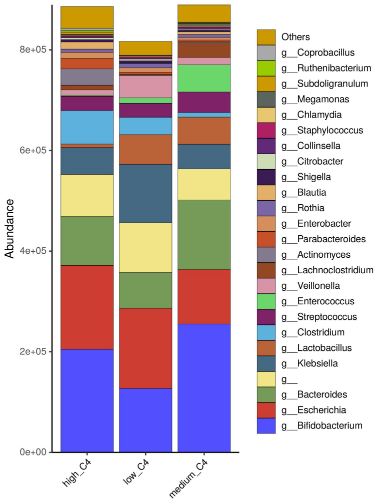

Abundance
0e+00
2e+05
4e+05
6e+05
8e+05
high_C4
low_C4
medium_C4
Others
g__Coprobacillus
g__Ruthenibacterium
g__Subdoligranulum
g__Megamonas
g__Chlamydia
g__Staphylococcus
g__Collinsella
g__Citrobacter
g__Shigella
g__Blautia
g__Rothia
g__Enterobacter
g__Parabacteroides
g__Actinomyces
g__Lachnoclostridium
g__Veillonella
g__Enterococcus
g__Streptococcus
g__Clostridium
g__Lactobacillus
g__Klebsiella
g__
g__Bacteroides
g__Escherichia
g__Bifidobacterium

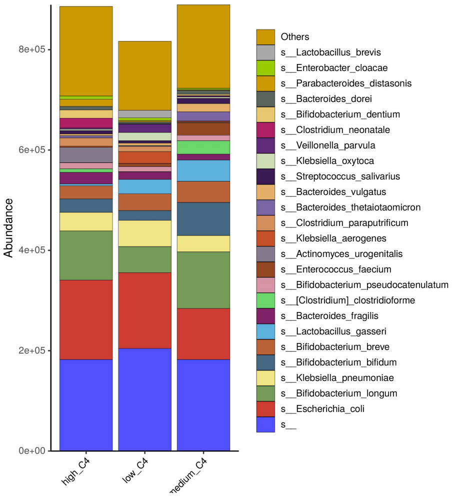

Abundance
0e+00
2e+05
4e+05
6e+05
8e+05
high_C4
low_C4
medium_C4
Others
s__Lactobacillus_brevis
s__Enterobacter_cloacae
s__Parabacteroides_distasonis
s__Bacteroides_dorei
s__Bifidobacterium_dentium
s__Clostridium_neonatale
s__Veillonella_parvula
s__Klebsiella_oxytoca
s__Streptococcus_salivarius
s__Bacteroides_vulgatus
s__Bacteroides_thetaiotaomicron
s__Clostridium_paraputrificum
s__Klebsiella_aerogenes
s__Actinomyces_urogenitalis
s__Enterococcus_faecium
s__Bifidobacterium_pseudocatenulatum
s__[Clostridium]_clostridioforme
s__Bacteroides_fragilis
s__Lactobacillus_gasseri
s__Bifidobacterium_breve
s__Bifidobacterium_bifidum
s__Klebsiella_pneumoniae
s__Bifidobacterium_longum
s__Escherichia_coli
s__

mC4

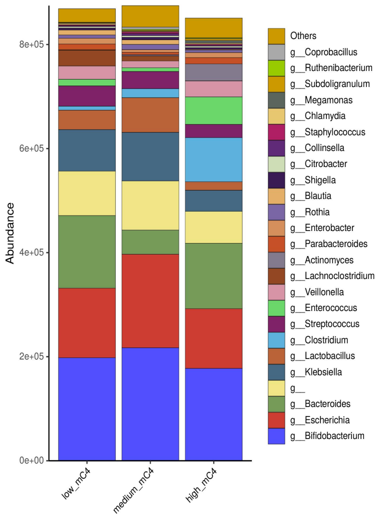

Abundance
0e+00
2e+05
4e+05
6e+05
8e+05
low_mC4
medium_mC4
high_mC4
Others
g__Coprobacillus
g__Ruthenibacterium
g__Subdoligranulum
g__Megamonas
g__Chlamydia
g__Staphylococcus
g__Collinsella
g__Citrobacter
g__Shigella
g__Blautia
g__Rothia
g__Enterobacter
g__Parabacteroides
g__Actinomyces
g__Lachnoclostridium
g__Veillonella
g__Enterococcus
g__Streptococcus
g__Clostridium
g__Lactobacillus
g__Klebsiella
g__
g__Bacteroides
g__Escherichia
g__Bifidobacterium

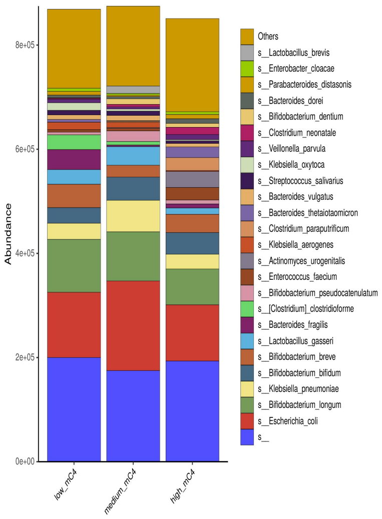

Abundance
0e+00
2e+05
4e+05
6e+05
8e+05
low_mC4
medium_mC4
high_mC4
Others
s__Lactobacillus_brevis
s__Enterobacter_cloacae
s__Parabacteroides_distasonis
s__Bacteroides_dorei
s__Bifidobacterium_dentium
s__Clostridium_neonatale
s__Veillonella_parvula
s__Klebsiella_oxytoca
s__Streptococcus_salivarius
s__Bacteroides_vulgatus
s__Bacteroides_thetaiotaomicron
s__Clostridium_paraputrificum
s__Klebsiella_aerogenes
s__Actinomyces_urogenitalis
s__Enterococcus_faecium
s__Bifidobacterium_pseudocatenulatum
s__[Clostridium]_clostridioforme
s__Bacteroides_fragilis
s__Lactobacillus_gasseri
s__Bifidobacterium_breve
s__Bifidobacterium_bifidum
s__Klebsiella_pneumoniae
s__Bifidobacterium_longum
s__Escherichia_coli
s__

C3

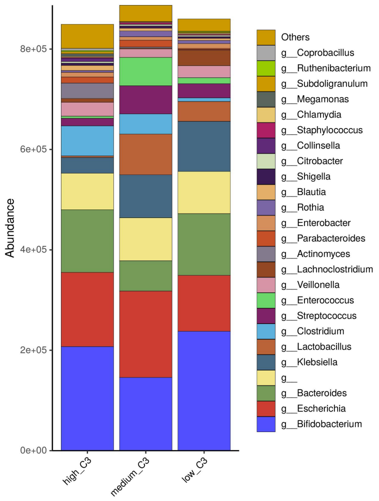

Abundance
0e+00
2e+05
4e+05
6e+05
8e+05
high_C3
medium_C3
low_C3
Others
g__Coprobacillus
g__Ruthenibacterium
g__Subdoligranulum
g__Megamonas
g__Chlamydia
g__Staphylococcus
g__Collinsella
g__Citrobacter
g__Shigella
g__Blautia
g__Rothia
g__Enterobacter
g__Parabacteroides
g__Actinomyces
g__Lachnoclostridium
g__Veillonella
g__Enterococcus
g__Streptococcus
g__Clostridium
g__Lactobacillus
g__Klebsiella
g__
g__Bacteroides
g__Escherichia
g__Bifidobacterium

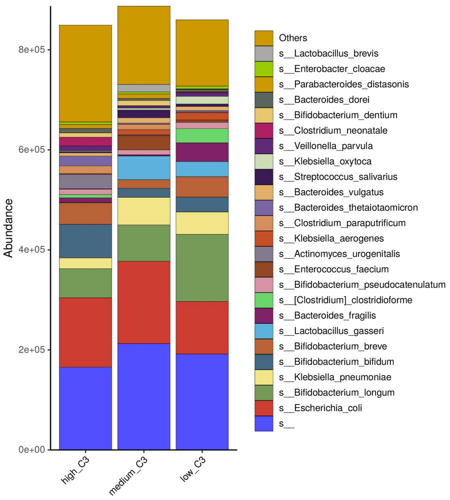

Abundance
0e+00
2e+05
4e+05
6e+05
8e+05
high_C3
medium_C3
low_C3
Others
s__Lactobacillus_brevis
s__Enterobacter_cloacae
s__Parabacteroides_distasonis
s__Bacteroides_dorei
s__Bifidobacterium_dentium
s__Clostridium_neonatale
s__Veillonella_parvula
s__Klebsiella_oxytoca
s__Streptococcus_salivarius
s__Bacteroides_vulgatus
s__Bacteroides_thetaiotaomicron
s__Clostridium_paraputrificum
s__Klebsiella_aerogenes
s__Actinomyces_urogenitalis
s__Enterococcus_faecium
s__Bifidobacterium_pseudocatenulatum
s__[Clostridium]_clostridioforme
s__Bacteroides_fragilis
s__Lactobacillus_gasseri
s__Bifidobacterium_breve
s__Bifidobacterium_bifidum
s__Klebsiella_pneumoniae
s__Bifidobacterium_longum
s__Escherichia_coli
s__

C2

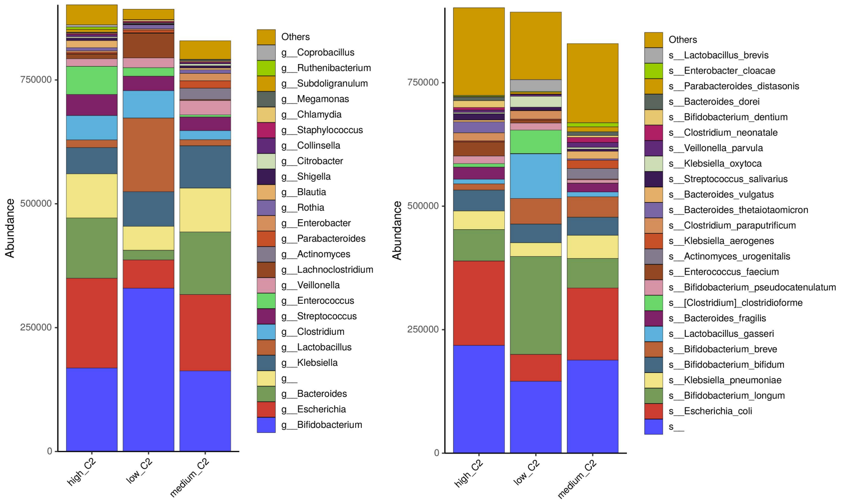

Supplementary Figure S5. The relative contributions of the top 25 genera and the species present in each group.
